# Comparison of Dengue Virus Type 2-Specific Small RNAs from RNA Interference-Competent and –Incompetent Mosquito Cells

**DOI:** 10.1371/journal.pntd.0000848

**Published:** 2010-10-26

**Authors:** Jaclyn C. Scott, Doug E. Brackney, Corey L. Campbell, Virginie Bondu-Hawkins, Brian Hjelle, Greg D. Ebel, Ken E. Olson, Carol D. Blair

**Affiliations:** 1 Arthropod-Borne and Infectious Diseases Laboratory, Department of Microbiology, Immunology and Pathology, Colorado State University, Fort Collins, Colorado, United States of America; 2 Center for Infectious Diseases and Immunity and Department of Pathology, University of New Mexico School of Medicine, Albuquerque, New Mexico, United States of America; The University of Queensland, Australia

## Abstract

The exogenous RNA interference (RNAi) pathway is an important antiviral defense against arboviruses in mosquitoes, and virus-specific small interfering (si)RNAs are key components of this pathway. Understanding the biogenesis of siRNAs in mosquitoes could have important ramifications in using RNAi to control arbovirus transmission. Using deep sequencing technology, we characterized dengue virus type 2 (DENV2)-specific small RNAs produced during infection of *Aedes aegypti* mosquitoes and *A. aegypti* Aag2 cell cultures and compared them to those produced in the C6/36 *Aedes albopictus* cell line. We show that the size and mixed polarity of virus-specific small RNAs from DENV-infected *A. aegypti* cells indicate that they are products of Dicer-2 (Dcr2) cleavage of long dsRNA, whereas C6/36 cells generate DENV2-specific small RNAs that are longer and predominantly positive polarity, suggesting that they originate from a different small RNA pathway. Examination of virus-specific small RNAs after infection of the two mosquito cell lines with the insect-only flavivirus cell fusing agent virus (CFAV) corroborated these findings. An *in vitro* assay also showed that Aag2 *A. aegypti* cells are capable of siRNA production, while C6/36 *A. albopictus* cells exhibit inefficient Dcr2 cleavage of long dsRNA. Defective expression or function of Dcr2, the key initiator of the RNAi pathway, might explain the comparatively robust growth of arthropod-borne viruses in the C6/36 cell line, which has been used frequently as a surrogate for studying molecular interactions between arboviruses and cells of their mosquito hosts.

## Author Summary

Understanding how arthropod-borne viruses (arboviruses) establish persistent infections in mosquitoes will help us to find ways to prevent viral disease transmission by these insects. RNA silencing pathways in mosquitoes and other insects, particularly RNA interference (RNAi), have been shown to be important in antiviral defense. In this study we describe small RNAs involved in RNA silencing that are derived from the genome of the arbovirus dengue virus type-2 (DENV2) in infected *Aedes aegypti* mosquito cell lines and mosquitoes. We also show that C6/36, a mosquito cell line from *A. albopictus*, appears to process DENV2 RNA for silencing differently from *A. aegypti* mosquitoes, revealing that other small RNA pathways in mosquito cells might have a role in antiviral immunity in this cell line and provide insight into using mosquito cell cultures to study the antiviral response to arboviruses in mosquitoes.

## Introduction

Mosquito cell cultures are used routinely in arbovirology studies to grow viruses and to elucidate aspects of viral infection and replication in mosquitoes. Many of these cell lines were established by Peleg and Singh in the 1960's [1], [2]. A mosquito cell line designated C6/36 resulted from a clone selected by Igarashi from Singh's *Aedes albopictus* larval line for its ability to grow dengue and chikungunya viruses to high titers [3], and has become one of the most commonly used of arbovirologists' tools [4], [5], [6], [7], [8]. Since *Aedes aegypti* is the most important vector for arboviruses such as dengue, we have used another cell line, derived from *A. aegypti* embryos and known as Aag2, in several recent studies [9], [10]. This cell line was originally established by Peleg in 1968 and was further characterized by Lan and Fallon in 1990 [11].

RNA interference (RNAi) has been shown to play an important role in insect antiviral immunity [12], [13], [14]. RNAi is a molecular pathway that is triggered by exogenous long double-stranded RNA (dsRNA) in the cytoplasm. Much of what we know about RNAi in insects has been elucidated in *Drosophila* flies and cultured cells. Dicer-2 (Dcr2) is a multi-domain RNase III that recognizes and cleaves dsRNA into small interfering RNAs (siRNAs) to initiate the RNAi pathway. The siRNAs are usually 21 bp in length with 5′ phosphates and two nt overhangs on the 3′ hydroxyl ends [15], [16], [17], [18], [19]. siRNAs, in association with Dcr2 and the dsRNA-binding protein R2D2, are loaded into a multi-protein RNA-induced silencing complex (RISC), which contains the endonuclease Argonaute-2 (Ago2) [20], [21], [22]. The RISC unwinds and degrades one of the siRNA strands, and retains the other strand for use as a guide to identify long single-stranded RNA (ssRNA), such as viral mRNA, which is complementary to the siRNA. In the effector phase of RNAi, Ago2 cleaves the long ssRNA at the point of complementarity, leading to its further destruction [23], [24], [25].

Other RNA silencing pathways, including Piwi-interacting (piRNA) and endogenous siRNA (endo-siRNA), have been discovered in *Drosophila* [26], [27], [28], [29], [30], [31], [32]. piRNAs associate with members of the Piwi clade of the Argonaute proteins, which includes Piwi, Aubergine (Aub) and Argonaute 3 (Ago3) in *Drosophila*. piRNAs are approximately 24–30 nt in length and are modified by DmHEN1 (also known as Pimet) by 2′-O-methylation on their 3′ termini [33], [34]. The piRNA pathway trigger appears to be single-stranded RNA since the small RNAs are almost always of a single polarity, and the biogenesis is Dcr1- and Dcr2-independent, possibly using the endonuclease activity of the Piwi proteins, at least in determining their 5′ ends [27], [28], [35]. The piRNA pathway is believed to have important roles in controlling the transcription of transposable elements in the genome and in development of reproductive tissues. Recently, virus-derived piRNAs were discovered in cultured *Drosophila* ovary somatic sheet cells [36].

Genomic analyses show that mosquitoes encode *Drosophila-*orthologous RNAi pathway components including Dcr2, R2D2, and Ago2, as well as paralogous microRNA (miRNA) pathway components Drosha, Pasha, Dicer-1 (Dcr1), Loquacious (Loqs; R3D1), and Argonaute-1 (Ago1) [37]. Deep sequencing analyses have characterized miRNAs in *A. aegypti* [38] and *Culex quinquefasciatus* [39] and have demonstrated altered levels of expression after blood-feeding [38] and after WNV infection [39].

Dengue virus serotypes 1-4 (DENV1-4; genus *Flavivirus*; family *Flaviviridae*) are the most important mosquito-borne viruses affecting humans. They are hyperendemic throughout the tropics and are transmitted primarily by *A. aegypti* mosquitoes in an urban cycle. The DENV genome is a single-stranded, positive-sense RNA approximately 10.7 kilobases (kb) in length with a 5′ cap structure, but no 3′ polyA tail. It encodes three structural proteins and seven non-structural proteins. Viral RNA replication occurs in the perinuclear region of the cytoplasm in membrane-enclosed replication complexes [40], [41]. During replication, a full-length negative-sense complementary RNA is used as a template for genome synthesis, resulting in a replicative form consisting of long viral dsRNA [42].

We recently showed that injection of *A. aegypti* mosquitoes with dsRNA derived from *A. aegypti dcr2* or *r2d2* mRNA to knock-down expression of RNAi pathway components, followed two days later by oral challenge with DENV2, resulted in increased virus titers in whole mosquitoes compared to non-injected or unrelated dsRNA (*β-gal*)-injected mosquitoes [10], indicating a role for Dcr2 in the mosquito antiviral response. DENV2-related si-like RNAs (viRNAs) were also detected in DENV2-infected *A. aegypti* and Aag2 cells in this study [10]. West Nile virus (WNV)-derived viRNAs were detected in WNV-infected *Culex* mosquitoes via deep sequencing [43]; however, small RNAs derived from WNV RNA were not detectable in northern blots from WNV-infected C6/36 cells [8]. We know of no other reports of detection or characterization of flavivirus-derived siRNAs.

RNAi has also been shown to be an important antiviral pathway in alphavirus infections of *A. aegypti* mosquitoes. Co-injection of dsRNA derived from *A. aegypti ago2* or *dcr2* mRNA with Sindbis virus (SINV) TR339-eGFP (genus *Alphavirus*; family *Togaviridae*) into *A. aegypti* resulted in increases in detectable viral RNA, infectious virus titers and infection rates of mosquitoes [44]. When SINV were engineered to express the B2 protein, a viral suppressor of RNAi, the viruses replicated to higher titers in mosquitoes and Aag2 mosquito cell cultures, and caused cytopathic effects in cell cultures and mortality in mosquitoes, suggesting the importance of the RNAi pathway in maintaining persistent, non-pathogenic arboviral infections of the mosquito host [9], [45]. RNAi as an antiviral defense was also demonstrated in *Anopheles gambiae* mosquitoes, in which injection of dsRNA to knock-down expression of Ago2 resulted in increased replication and dissemination of another alphavirus, O'nyong-nyong virus (ONNV) [46]. ONNV-derived viRNAs were identified in *A. gambiae* mosquitoes by deep sequencing [45].

Despite the demonstration of antiviral RNAi in mosquitoes and mosquito cells, arboviruses are able to establish persistent, generally non-cytopathic infections in their natural vectors. Furthermore, the mechanistic details of small RNA production have not been confirmed in mosquitoes or mosquito cell lines. Since we have observed more robust growth of DENV2 in C6/36 than in Aag2 cells, we hypothesized that this difference was due to variations in the RNAi responses of the two cell lines. We describe here a comparative study of DENV2-specific small RNAs made during infection of Aag2 and C6/36 mosquito cell cultures and *A. aegypti* mosquitoes, and present evidence that C6/36 cells have an aberrant antiviral RNAi pathway.

## Materials and Methods

### Viruses and cell cultures

The DENV2 strain used for infections of cells and mosquitoes was highly passaged Jamaica 1409. DENV2 stocks used for infection of C6/36 cells and Aag2 cells were propagated in Aag2 cells. Because the Aag2 cell line is persistently infected with the mosquito-only flavivirus cell fusing agent virus (CFAV), DENV2 stocks contained infectious CFAV. C6/36 (*A. albopictus*) cells were grown in L-15 medium with 10% fetal bovine serum (FBS), 100 U/ml/100 µg/ml penicillin/streptomycin (P/S), and L-glutamine (L-glut) at 28°C (without CO_2_). C6/36 cell infections were done in L-15 with 2% FBS, P/S, L-glut and non-essential amino acids (NEAA) at 28°C (without CO_2_). Aag2 (*A. aegypti*) cells were grown in Schneider's *Drosophila* medium with 10% FBS, P/S, and L-glut at 28°C (without CO_2_). Aag2 cell infections were done in Schneider's *Drosophila* medium with 2% FBS, P/S, L-glut and NEAA at 28°C (without CO_2_). Cells were infected with DENV2 at a multiplicity of infection (MOI) of 0.1, and cell RNA was harvested at one and five days post DENV2- or mock- infection. LLC-MK2 monkey kidney cells were cultured in modified Eagle's medium (MEM) supplemented with 8% FBS, L-glut, NEAA and P/S and maintained at 37°C in the presence of CO_2_.

For the transfection experiments, Aag2 cells were grown as described above, while the C6/36 cell line was grown in MEM with 10% FBS, P/S, L-glut, and 0.015% sodium bicarbonate at 28°C in the presence of CO_2_.

### Infectious virus titration by plaque assay

LLC-MK2 cells were grown to confluent monolayers in 24-well plates, infected with 10-fold serial dilutions of virus for 1 hour and overlaid with an agarose-nutrient mixture. After 7 days incubation at 37°C cells were stained with 5 mg/ml MTT (3-[4,5-dimethylthiazol-2-yl]-2,5-diphenyltetrazolium bromide) solution. Viral titers were determined by counting plaques [9].

### Mosquitoes and DENV2 infection

*A. aegypti* RexD strain mosquitoes were reared at 28°C with 82% humidity. Female mosquitoes one-week post-eclosion were deprived of a sugar source overnight and were then allowed to feed on artificial bloodmeals containing defibrinated sheep blood (40%) (Colorado Serum Company, Boulder, CO) and an infected C6/36 cell suspension (60%) with 1 mM ATP for one hour. The bloodmeal was maintained at 37°C in a water-jacketed glass feeder covered with hog gut membrane, and mosquitoes fed on the blood through the membrane. The bloodmeal titer of DENV2 strain Jamaica 1409 was approximately 1×10^7^ PFU/ml, while the mock-infected mosquitoes were given a blood and uninfected C6/36 cell mixture. Bloodfed females were selected and were maintained with water and sugar for nine days after the infection (or mock infection), when RNA was harvested from 20 whole mosquitoes per group.

### Preparation of dsRNA substrate for *in vitro* dicing assay and RNA silencing

dsRNA was prepared by *in vitro* transcription from a PCR product of a 498 bp region of the *E. coli* beta-galactosidase (β-gal) gene with T7 promoters on both strands. Transcription was carried out using the Megascript T7 Kit (Applied Biosystems, Foster City, CA) for approximately 16 hours at 37°C with approximately 9% of the UTP substrate conjugated to biotin (Applied Biosystems). The reaction mixture was treated with Turbo DNase (Applied Biosystems) for 30 minutes, followed by a phenol/chloroform extraction and an overnight ethanol precipitation. The RNA was fractionated on a TBE-urea 6% polyacrylamide gel (Invitrogen, Carlsbad, CA) and small RNA was eluted overnight at room temperature. RNA was extracted with phenol/chloroform (5∶1), followed by chloroform/isoamyl alcohol (24∶1), and precipitated overnight at −20°C in ethanol. The RNA was quantified by spectrophotometry.

### *In vitro* dicing assay

Cell-free lysates were generated from Aag2 cells and C6/36 cells using a previously described protocol [47]. Briefly, cells were washed in PBS three times, then resuspended in 1X lysis buffer with protease inhibitors and 5 mM DTT. The cells were disrupted in a Dounce homogenizer, and then centrifuged at 14,000 rpm for 25 minutes at 4°C. The supernatant was flash frozen in a dry ice/ethanol bath and stored at −80°C. Protein concentrations were determined with the DC Protein Assay (Bio-Rad Laboratories Inc., Hercules, CA) and samples were equilibrated to the same protein concentration using lysis buffer immediately before the dicing assay was set up. Dicing activity reactions contained 1/2 volume lysate, 1/3 volume 40X reaction mix and approximately 70 nanograms of 498 bp biotinylated β-gal dsRNA, with the lysate being added last. At each timepoint, 10 microliters (µl) of the reaction were removed, added to 2X PK buffer and flash frozen. RNA was extracted using phenol/chloroform (5∶1), followed by chloroform/isoamyl alcohol (24∶1), and precipitated overnight at −20°C in ethanol. RNA was electrophoresed on a TBE non-denaturing 20% polyacrylamide gel (Invitrogen), electrophoretically transferred to a positively charged nylon BrightStar-Plus membrane (Applied Biosystems) and UV-crosslinked to the membrane. Biotinylated RNA was detected with the BrightStar BioDetect Kit (Applied Biosystems) and exposed to autoradiography film. In some reactions, 1 µl (0.5 unit) of recombinant human dicer enzyme (Genlantis Inc., San Diego, CA) was added to the 10 µl reaction just before addition of the lysate.

### Plasmid construction

The enhanced green fluorescent protein (EGFP) gene was amplified from the pEGFP-1 plasmid (Clontech, Mountain View, CA) using the forward primer EGFP-*Nco* I F and reverse primer EGFP-*Xho* I R. The amplicon was digested with *Nco* I and *Xho* I and cloned into the insect-specific expression plasmid pIEx (Novagen, Madison, WI) to generate the pIEx-EGFP vector.

### Small interfering RNAs and double stranded RNAs

The Accell EGFP siRNA used in these experiments and the control WNV siRNA were synthetically produced by Dharmacon (Lafayette, CO). WNV siRNA was complementary to a 21 nt region of the WNV genome starting at position 85 in the capsid gene.

Approximately 500 bp fragments corresponding to EGFP or WNV capsid genes were amplified using primers that included a T7 promoter sequence in both the forward and reverse primers. The amplicons were PCR purified and subsequently used as templates for dsRNA transcription. Synthesis of dsRNA molecules was carried out using the T7 Megascript kit (Applied Biosystems) as described above with omission of biotinylated UTP. The dsRNA was re-suspended in 50 µl PBS, quantified and brought to a final concentration of 1 µg/µl.

### Transfection Conditions

The day prior to transfection, Aag2 or C6/36 cells were seeded in 24-well tissue culture plates at a density of 5×10^5^ cells/well. For the transfections, 250 ng/well of the pIEx-EGFP plasmid were combined with either EGFP or WNV siRNA (to a final concentration of 50 nM), or 1 µg/well of EGFP or WNV dsRNA in Opti-MEM medium. Subsequently, the Attractene Transfection Reagent (Qiagen, Valencia, CA) was added and lipid-nucleic acid complexes were allowed to form for 15 min. at room temperature. The medium on the cells was discarded and 440 µl of Opti-MEM were added to each well followed by dropwise addition of 60 µl of the complexes. The cells remained in the presence of the transfection reagent for four hours, after which appropriate medium for each cell line was replaced. Cell viability was monitored for 48 hours post transfection, when cell images were acquired and the cells harvested. The cell pellets were re-suspended in 500 µl Trizol (Invitrogen) and total protein for immunoblots precipitated according the manufacturer's instructions.

### Microscopy

Images were acquired using a Nikon TE2000 inverted microscope with a Hamamatsu Orca camera and Wasabi software (Hamamatsu Photonics, Japan). Representative areas as determined by cell density were photographed under 10× magnification. Fluorescent images were acquired using a 222 ms exposure without gain and the light images were acquired using a 30 ms exposure without gain. The monochrome images were subsequently pseudo-colored using the Slidebook software (Intelligent Imaging Innovations, Denver, CO).

### Immunoblots

Total protein recovered from transfected cell cultures was quantified using the Bradford Kit on the Bio-Rad SmarSpec Plus spectrophotometer (Bio-Rad Laboratories Inc., Hercules, CA). Fifteen micrograms of total protein were separated on 12.5% SDS-PAGE and transferred to a nitrocellulose membrane. The presence of EGFP was detected using a primary mouse anti-*Aequorea victoria* EGFP monoclonal antibody (Clontech) at a dilution of 1∶1000 in TBST +5% non-fat dry milk. The blot was subsequently probed with phosphatase labeled goat anti-mouse IgG at a 1∶1000 dilution (KPL Inc., Gaithersburg, MD). Detection of actin was performed with primary rabbit polyclonal antibodies at a 1∶1000 dilution in TBST +5% BSA (Abcam, Cambridge, MA) and phosphatase labeled goat anti-rabbit IgG secondary antibody at a 1∶1000 dilution (KPL Inc.). Membranes were developed with the 1-Step NBT/BCIP reagent for 5–10 minutes at room temperature (Pierce, Rockford, IL).

### Small RNA isolation and library preparation

Total RNA was extracted using TRIzol (Invitrogen) with manufacturer's instructions from Aag2 and C6/36 cells mock- or DENV2-infected (MOI = 0.1) at 5 days post infection. Total RNA was extracted with TRIzol from non-infectious bloodfed and DENV2 bloodfed *A. aegypti* mosquitoes at nine days post bloodmeal. Small RNA was isolated using the FlashPAGE Fractionator (Applied Biosystems). Small RNA libraries were made using SOLiD small RNA expression kit (Applied Biosystems) and were sequenced at the University of Washington on a SOLiD sequencer (Applied Biosystems).

### viRNA sequencing analysis

Potential viRNAs were aligned to the DENV2 or CFAV genome using NextGENe software (Softgenetics, LLC, State College, PA), Version 1.11, running the transcriptome assembly function. CSFASTA (color-space) files from SOLiD sequencing of samples were used as the sample file and a FASTA file of either DENV2 Jamaica 1409 RNA from Genbank accession number M20558.1 or CFAV RNA Genbank accession number NC001564.1 was used as the reference sequence.

### Logo analysis

Logo analysis was performed using WebLogo 3 located at http://weblogo.threeplusone.com in March, 2010 [48], [49]. Reads that matched DENV2 and CFAV genomes were identified with NextGENe alignment, converted to base-space with NextGENe, and used in the WebLogo. The full length (35 nt) of the matched read was used to allow comparison of all of the viRNAs at once as all reads must be the same length when analyzed with the WebLogo program. The program default settings were used, except the Y-axis scale was set to 1 bit.

### Dicer-2 northern blot hybridization

5 µg of total RNA from Aag2 or C6/36 cell cultures was heated at 95°C for 5 minutes, placed on ice, then loaded onto a 1.25% denaturing formaldehyde agarose gel and electrophoresed in MOPS/formaldehyde buffer. The RNA was passively transferred from the gel overnight to a BrightStar-Plus positively charged nylon membrane (Applied Biosystems) with 10X SSC buffer. The membrane was autocrosslinked twice and pre-hybridized in 5 ml of UltraHyb Hybridization Buffer (Applied Biosystems) for 1 hour at 68°C. Biotinylated *dcr2* antisense ssRNA probes (nt 4919-5116) were added to the hybridization buffer to a final concentration of 0.1 nM (approximately 80 ng of probe in 5 ml buffer). *A. aegypti dcr2* probe was added to the membrane with the Aag2 RNA bound, and *A. albopictus* dcr2 probe was added to the membrane with C6/36 RNA bound and the hybridizations took place in separate tubes. Membranes and probes hybridized for 18 hours at 68°C. Membranes were then washed twice for 30 minutes in 2X SSC, 0.1% SDS buffer and twice for 60 minutes in 0.1X SSC, 0.1% SDS buffer. All washes were done at 68°C and *A. aegypti* and *A. albopictus* membranes remained in separate tubes. The biotinylated probes attached to the membrane were detected with the BrightStar BioDetect Kit (Applied Biosystems), following manufacturer's instructions, with all washes performed for maximum recommended times. The membranes were exposed to autoradiography film for various times and the film was developed in an automatic autoradiography developer. Band intensities were compared with the BioRad Quantity One software, with adjustment for background.

## Results

### Characterization of DENV2-derived small RNAs

**Figure 1:**
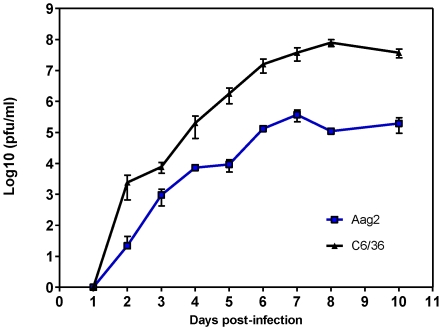
DENV2 strain Jamaica 1409 grows to higher titers in C6/36 than in Aag2 mosquito cell lines. Cell cultures were infected at a MOI of 0.001 and aliquots of medium were removed at 24 hour intervals and titrated by plaque assay. The assays were performed in triplicate and titers are expressed as means ± SEM.

DENV2-specific small RNAs from either mock- or DENV2-infected Aag2 cells, C6/36 cells, or *A. aegypti* mosquitoes were sequenced with the ABI SOLiD 2 sequencer and analyzed using NextGENe software. The small RNA library from DENV2-infected Aag2 cells at five days post-infection contained 1,612 viRNAs that aligned to the DENV2 genome from over 12×10^6^ reads (Table 1). Although this is a much higher number than in the uninfected or DENV2-infected cells at 1 dpi, it accounted for only 0.01% of the total number of small RNA reads from the library, possibly due to low levels of viral replication, or to sequestration of the dsRNA trigger in cellular membrane-enclosed vesicles [40], [41], [50]. The DENV2-infected *A. aegypti* mosquito library had 6,029 DENV2-specific small RNAs at 9 dpi, accounting for only 0.05% of the total small RNA reads. Many more DENV2-specific small RNAs (24,938 from over 12×10^6^ reads) were found in the C6/36 cells at 5 dpi (Table 1); this is possibly related to the ability of DENV2 to grow to 10- to 100-fold higher titers in these cells than in Aag2 cells (Figure 1).

**Table 1:** DENV2 viRNAs from whole mosquito and mosquito cell culture libraries.

Sample(Total # reads)	DENV2 viRNAs (% total)	%positive sense	%negative sense
Aag2 Mock(14,087,714)	93 (7×10^−4^%)	17%	83%
Aag2 DENV 1dpi(12,615,439)	55 (4×10^−4^%)	41%	59%
Aag2 DENV 5dpi(12,131,018)	1612 (0.01%)	59%	41%
Mosquito Mock(7,687,058)	30 (4×10^−4^%)	60%	40%
Mosquito DENV2 9dpi(12,267,708)	6029 (0.05%)	55%	45%
C6/36 Mock(11,915,311)	57 (5×10^−4^%)	61%	39%
C6/36 DENV2 5dpi(12,558,261)	24938 (0.2%)	96%	4%

The DENV2-specific small RNAs from Aag2 cells at 5 days post DENV2-infection were 59% positive (genome) sense, and the small RNAs from DENV2-infected mosquitoes were 55% positive sense (Table 1). Nearly-equal ratios of positive to negative sense DENV2 small RNAs suggested that most small RNAs are derived from dsRNA replicative intermediates, rather than intrastrand secondary structures in the ssRNA genome. In C6/36 cells, DENV2-specific small RNAs were 96% positive sense, suggesting that they were derived from ssRNA, and were not generated by Dcr2 cleavage of dsRNA as in *A. aegypti* cell cultures and mosquitoes.

**Figure 2:**
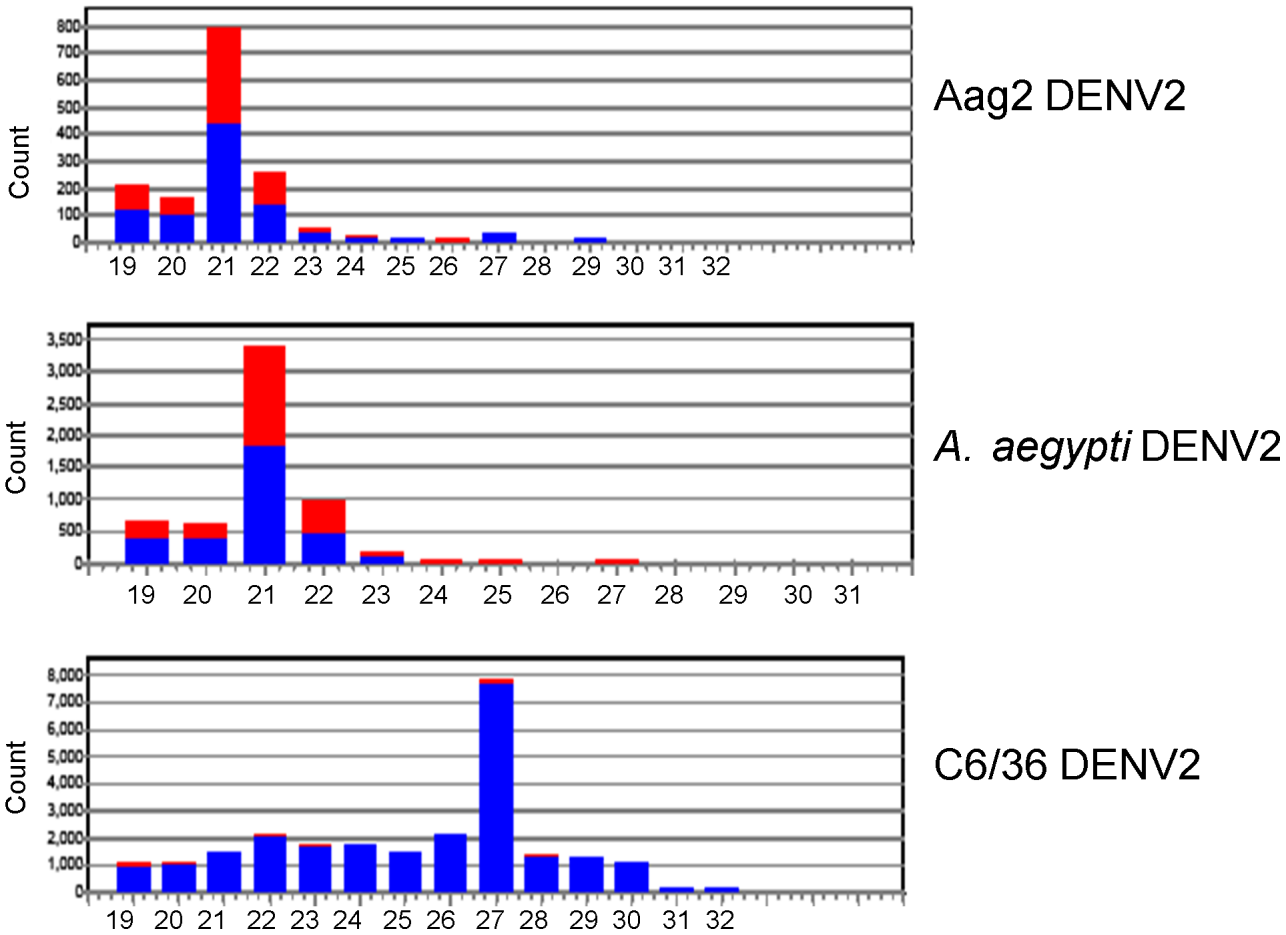
viRNA size distribution varies among DENV2-infected Aag2 and C6/36 cell cultures and *Aedes aegypti* mosquitoes. Shown are Aag2 (5 dpi) library, C6/36 (5 dpi) library, *A. aegypti* (9 dpi) library. Red bars, negative-sense viRNAs; blue bars, positive-sense viRNAs. Note differences in Y-axes among graphs.

The predominant size of DENV2-specific small RNAs in the Aag2 library (5 dpi) and in the DENV2-infected *A. aegypti* mosquitoes was 21 nt, which is the expected size for Dcr2 products, confirming that the exogenous-siRNA pathway was the most likely mechanism used by these cells to target DENV2 dsRNA (Fig. 2). The most common size of DENV2 small RNAs in the C6/36 cell library was 27 nt, which is not expected from the exogenous siRNA pathway. Furthermore, the few 21 nt DENV2-derived RNAs in the C6/36 cell library were predominantly positive sense, unlike the more nearly equal sense to antisense ratio found in the Aag2 cell library.

**Figure 3:**
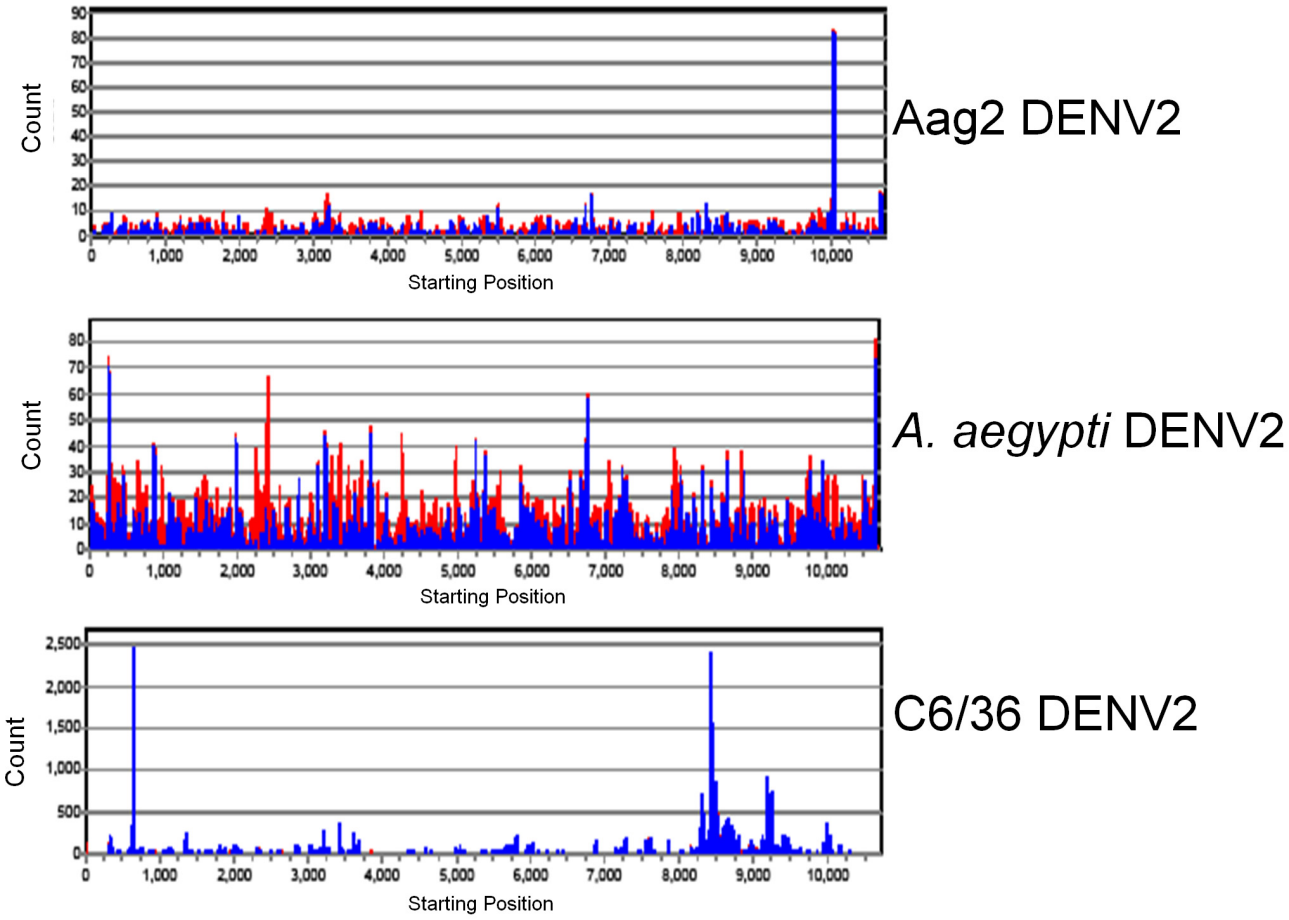
viRNA genome coverage distribution varies among DENV2-infected Aag2 and C6/36 cell cultures and *Aedes aegypti* mosquitoes. viRNA coverage across DENV genome for each library. Shown are Aag2 (5 dpi) library, C6/36 (5 dpi) library, *A. aegypti* (9 dpi) library. Red bars, negative-sense viRNAs; blue bars, positive-sense viRNAs. Note differences in Y-axes among graphs.

The sequences of the DENV2-specific small RNAs in the Aag2 cell library (5 dpi) were distributed evenly across the entire DENV2 genome, with the exception of a higher proportion of reads from one site around 10,000 nt (Fig. 3), further indicating that dsRNA replicative intermediates were the target of Dcr2 cleavage. The distribution along the viral genome of the DENV2 viRNAs from infected mosquitoes was somewhat different from that seen in the DENV2-infected Aag2 cells (Fig. 3), with several ‘hot spots’ for origins of either positive-sense or negative-sense viRNA, suggesting that ssRNA secondary structures in the DENV2 genome or its complement may also have been targeted by Dcr2, and to a greater extent in the mosquito than in cell culture. Analysis of potential secondary structures in the DENV2 RNA genome using mFold (www.bioinfo.rpi.edu/applications/mfold) predicts optimal-energy RNA configurations with extensive intrastrand base-pairing throughout the genome; however, most of these dsRNA structures lack perfect base-pairing over regions >21 bp so it is difficult to correlate them with siRNA “hot-spots”. The DENV2-specific small RNAs in C6/36 cells were not equally distributed, but instead were derived from a few specific regions of the genome, which might represent intrastrand secondary structure in the positive-strand virus genome.

From over 14×10^6^ reads, there were only 93 matches to the DENV2 genome in the uninfected Aag2 cell sample (approaching 0% of total reads) (Table 1). The Aag2 library from 1 day post-DENV2 infection also had a very low number of DENV2-specific reads (55), suggesting that viral replication to produce the dsRNA trigger in the cell was at a low level early after infection.

### Determination of cultured mosquito cell dicing activity by *in vitro* and whole cell assays

**Figure 4:**
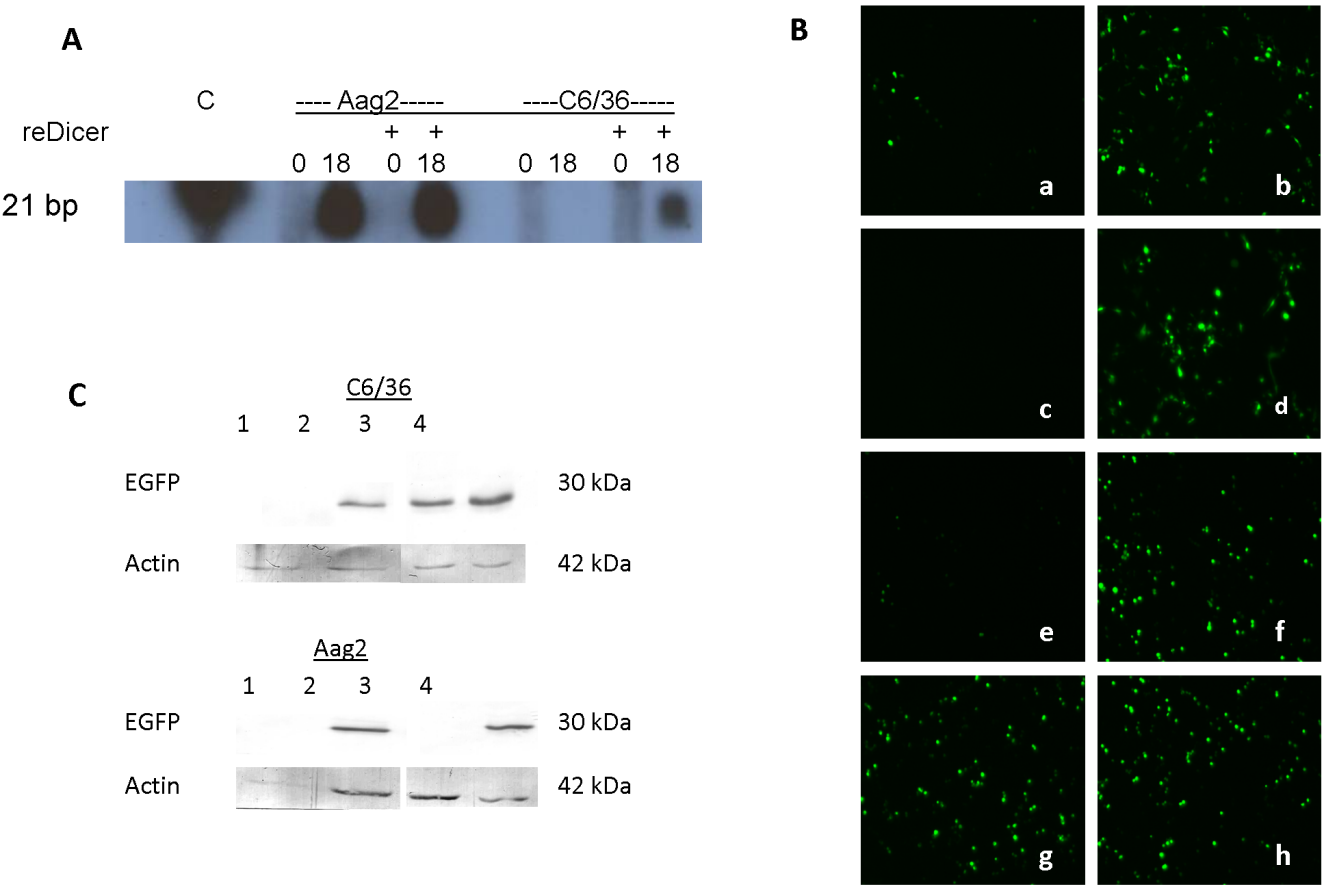
C6/36 cells are unable to produce a Dicer-2-like product. A) *In vitro* dicing assay. Biotinylated 500 bp β-gal dsRNA was added to Aag2 and C6/36 cell lysates, with (as indicated by +) or without human recombinant Dicer (reDicer), and aliquots were collected immediately (0) or after 18 hours. Lysate RNA was separated by electrophoresis on a polyacrylamide gel and transferred to a membrane for detection using the BrightStar detection system. C lane contains product of in vitro reaction of reDicer with 500 bp dsRNA, as size marker. (B) Analysis of dicing activity in Aag2 (a-d) and C6/36 (e-h) cell cultures. Aag2 cells were transfected with (a) pEGFP + EGFP siRNA, (b) pEGFP + WNV siRNA, (c) pEGFP + EGFP dsRNA, (d) pEGFP + WNV dsRNA. C6/36 cells were transfected with (e) pEGFP + EGFP siRNA, (f) pEGFP + WNV siRNA, (g) pEGFP + EGFP dsRNA, (h) pEGFP + WNV dsRNA. All cells were analyzed by fluorescent microscopy at 48 hr after transfection. (C) Immunoblot shows EGFP protein silencing. 1, pEGFP + EGFP siRNA, 2, pEGFP + WNV siRNA, 3, pEGFP + EGFP dsRNA, 4, pEGFP + WNV dsRNA.

We developed an *in vitro* dicing activity assay for cultured mosquito cells based on similar methods used to prepare *Drosophila* cell lysates [47]. This assay was used to compare the ability of cytoplasmic preparations from Aag2 and C6/36 cells to cleave a long exogenous dsRNA into 21 bp small RNAs, indicative of Dcr2 activity. Aag2 cell preparations produced the appropriate size product (matching the recombinant human dicer control product) within 18 hr after 500 bp long dsRNA was added to the lysate. C6/36 cell lysates did not make a 21 nt small RNA product from this labeled dsRNA during the same time period. When human recombinant dicer was added to the lysates, a siRNA-like product was made in the C6/36 cell lysate, indicating that although the C6/36 lysate lacks endogenous Dcr2 activity, it does not inhibit exogenously provided enzyme (Fig 4A).

Intact C6/36 and Aag2 cells were also tested for the ability of their RNAi pathways to inhibit EGFP expression from a transfected plasmid. Each cell line was transiently transformed with a plasmid expressing EGFP along with siRNAs or long dsRNA derived from the EGFP sequence or control RNAs derived from the WNV genome sequence. Transfection of EGFP-derived-siRNAs into either cell type resulted in knock-down of EGFP expression, indicating that both have a functional RNAi pathway if pre-formed siRNA is loaded into RISC. However, transfection of cognate long dsRNA resulted in knock-down of EGFP expression only in Aag2 cells, suggesting that only this cell line was able to efficiently carry out Dcr2-mediated cleavage of dsRNA (Fig 4B). Immunoblotting of fractionated cell proteins with EGFP antibodies confirmed the corresponding protein expression levels (Fig 4C). These results provide further evidence that C6/36 cells are defective in Dcr2 activity and suggest that both cell lines are able to form a functional RISC.

### CFAV small RNAs identified from SOLiD sequencing

During preliminary small RNA analysis, we detected cell fusing agent virus (CFAV) small RNA in Aag2 cells, suggesting persistent infection by this insect-only flavivirus. The sequences of small RNA libraries prepared from Aag2 cells were aligned with a CFAV genome sequence from GenBank as reference using NextGENe software. Surprisingly, there were many more small RNAs in all Aag2 libraries that aligned to CFAV RNA than to DENV2 RNA (Table 2). CFAV was first described in the precursor cell line to Aag2 cells [51], [52]. Neither the mock-infected nor DENV2-infected *A. aegypti* mosquitoes appeared to have a CFAV infection, as only a small number of reads from those libraries matched the CFAV genome (data not shown). The mock infected C6/36 cells also had <60 CFAV-specific small RNAs, but >21,000 CFAV small RNAs were detected in the C6/36 cell culture library 5 days post DENV2-infection (Table 2). Since the DENV2 stock used to infect the C6/36 cells was grown in Aag2 cells, this was probably the source of the CFAV, which was introduced to the C6/36 cells during the DENV2 infection.

**Table 2:** CFAV viRNAs from mosquito cell culture libraries.

Sample(Total # reads)	CFAV viRNAs(% total)	%positive sense	%negative sense
Aag2 Mock(14,087,714)	79,732 (0.6%)	57	43
Aag2 DENV2 Day 1(12,615,439)	25,663 (0.2%)	63	37
Aag2 DENV2 Day 5(12,131,018)	88,863 (0.7%)	54	46
C6/36 Mock(11,915,311)	59 (5×10^−4^%)	54	46
C6/36 DENV2 Day 5(12,558,261)	21,807 (0.2%)	99	1

**Figure 5:**
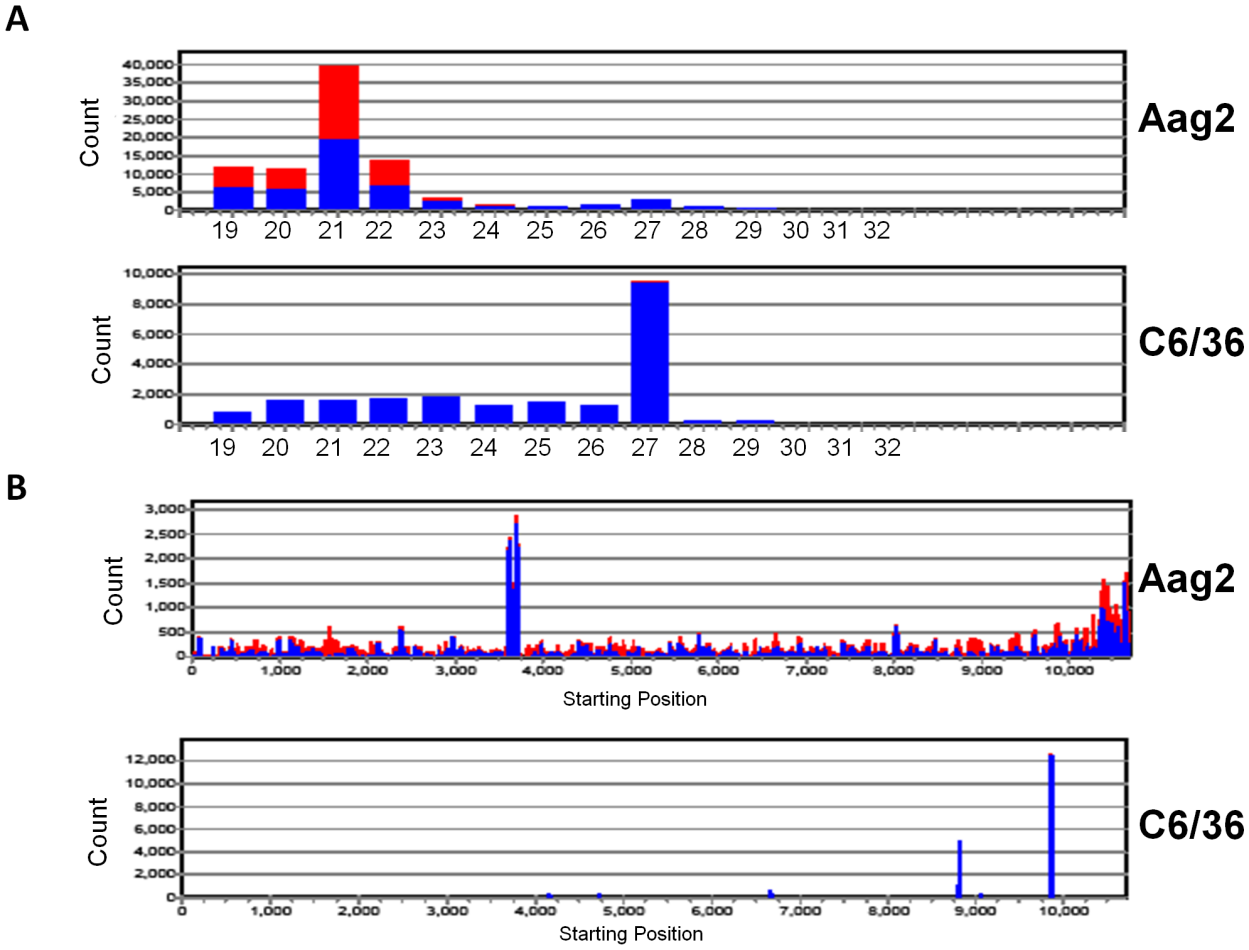
viRNA size and genome coverage distributions vary among CFAV-infected Aag2 and C6/36 cell cultures. (A) viRNA size distribution. (B) viRNA coverage across CFAV genome for each library. Shown are Aag2 (5dpi) library, C6/36 (5dpi) library. Red bars, negative-sense viRNAs; blue bars, positive-sense viRNAs. Note difference in Y-axes among graphs.

Interestingly, the patterns of size, polarity, and genome distribution of the CFAV-derived small RNAs were very similar to those of the DENV2-derived small RNAs in both cell lines (Table 2, Fig. 5A and B). Aag2 cell CFAV-specific small RNAs were predominantly 21 nt in length and were 54–63% positive sense. C6/36 cell CFAV-specific small RNAs were mostly 27 nt in length and 99% were derived from the positive sense strand, similar to the characteristics observed for DENV2-specific small RNAs, indicating that the defect in Dicer activity was not limited to production of DENV2 small RNAs.

### Logo Analysis of virus-specific small RNAs

**Figure 6:**
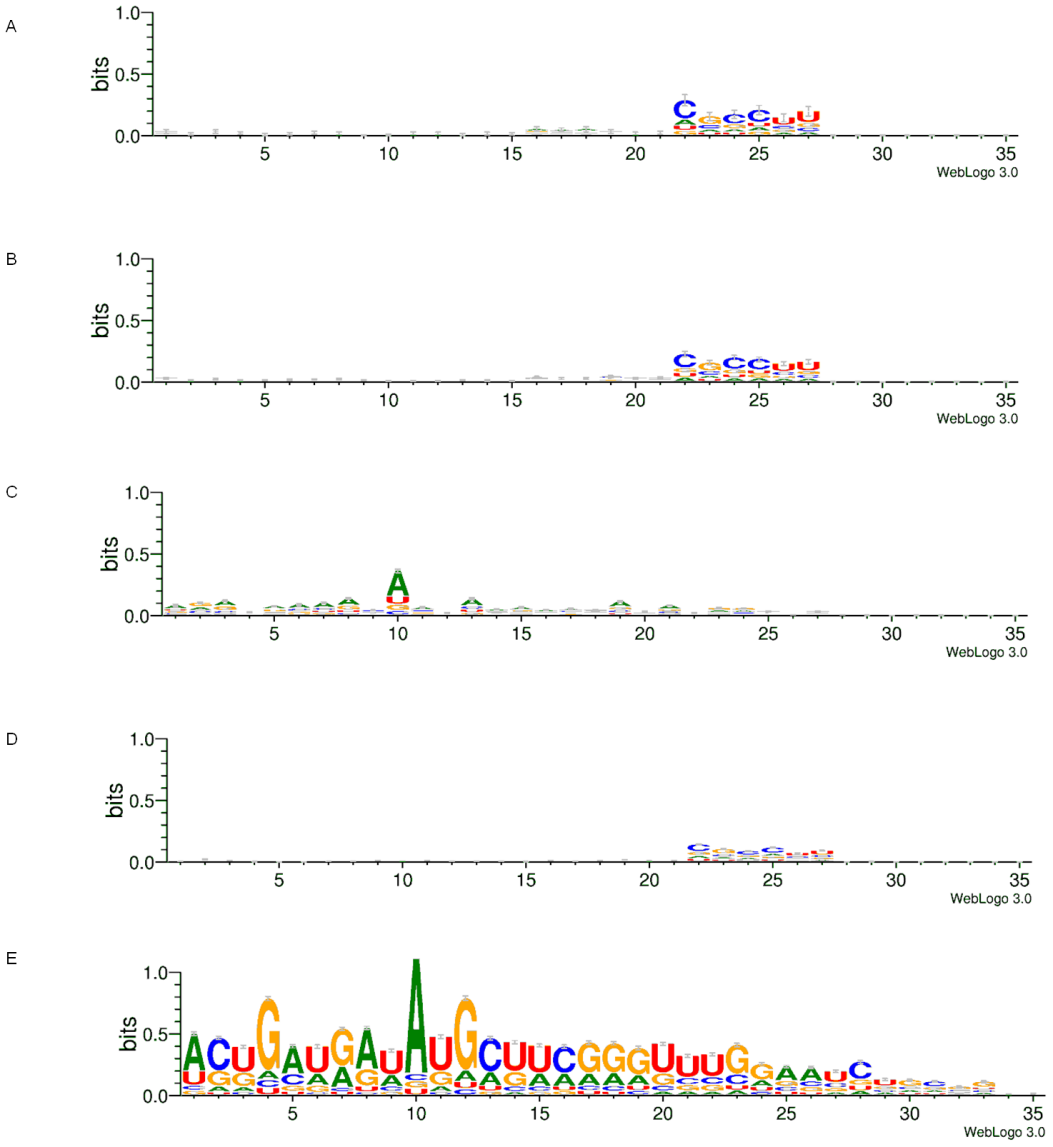
Logo analysis of DENV2 viRNA from mosquitoes and cell culture and CFAV viRNAs from cell culture. Logo analysis was performed on DENV2 and CFAV viRNAs using Weblogo 3. (A) Aag2 DENV2 (5 dpi) viRNA logo. (B) *A. aegypti* DENV2 (9 dpi) viRNA logo. (C) C6/36 DENV2 (5 dpi) viRNA logo. (D) Aag2 CFAV viRNA logo. (E) C6/36 CFAV viRNA logo.

The DENV2- and CFAV-specific small RNAs from both cell types were analyzed with the WebLogo 3 program (http://weblogo.threeplusone.com) to determine if there were preferences for specific nucleotides at certain positions. The total untrimmed 35 nt length of virus RNA-matching reads was analyzed in the program; therefore, the sequences for the six 3′-terminal nucleotides match the linker attached to the small RNAs in preparation of libraries. In both the DENV2 and CFAV viRNAs from Aag2 cells, there were no apparent preferences for specific nucleotides at any positions in the 5′ 21 nt. However, in the C6/36 cell libraries, there appeared to be a bias for adenine on the nucleotide at position 10 in both the DENV2-specific and CFAV-specific small RNAs (Fig 6). Ago3-associated Piwi-interacting RNAs (piRNAs) often have an adenine at the 10^th^ position, hinting at a possible mechanism for generation of these small RNAs in C6/36 cells [35], [53].

### Dcr2 expression in Aag2 and C6/36 cells

**Figure 7:**
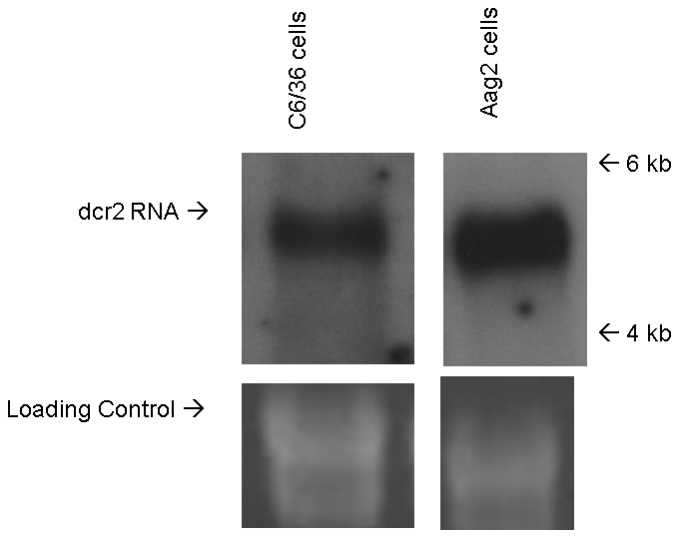
Northern blot hybridization to detect expression of *dicer2* mRNA in cultured mosquito cells. Total RNA from Aag2 or C6/36 cell cultures was fractionated by agarose gel electrophoresis, transferred to a nylon membrane, and hybridized to either an *A. albopictus dcr2* probe (left) or an *A. aegypti dcr2* probe (right). The biotinylated probes were detected with the BrightStar BioDetect Kit.

To examine levels of *dcr2* expression in C6/36 cells as a possible explanation for defective Dcr2 activity, we compared *dcr2* mRNA in Aag2 and C6/36 cells by northern blot hybridization of total RNA, using specific probes based on sequences of cDNA amplified from each cell line (data not shown). *dcr2* messenger of the anticipated size was detected in both Aag2 cells and C6/36 cells using their respective probes (Fig 7). Neither probe hybridized to RNA from the heterologous species (not shown). Comparison of band intensities determined that the Aag2 cell band was approximately 1.7-fold stronger than the C6/36 cell band.

## Discussion

The aims of this study were to further define the mechanisms of antiviral RNA silencing in mosquito cells infected with DENV2 by characterization of the virus-specific small RNAs (viRNAs) produced during infection and to test the hypothesis that enhanced virus production in C6/36 cells as compared to Aag2 cells is attributable to a less effective RNAi response in the former. We present further evidence that the RNAi response initiated by Dcr2 is central to antiviral defense in *A. aegypti* and that defective Dcr2 activity in C6/36 cells renders them less able to control DENV2 replication.

Little was previously known about the nature of the DENV2 RNA trigger of the RNAi antiviral pathway and the characteristics of resulting DENV2-specific siRNAs during the natural transmission cycle in mosquitoes. We previously reported enhancement of DENV2 replication after knock-down of *dcr2* expression and presence of virus-specific small RNA in *A. aegypti* [10], but our attempts to characterize these small RNAs using traditional cDNA cloning and sequencing methods yielded very few genome matches (unpublished); thus in this study we employed deep sequencing and analysis of small RNA libraries. Since DENV2 induces the production of dsRNA during its replication cycle [10], [42], [54], this would be the most obvious target for Dcr2 cleavage and activation of an RNAi response. Analysis of our deep sequencing data showed that 54–60% of the DENV2-small RNAs in Aag2 cells were positive sense, close to the 1∶1 ratio that would be expected if the trigger were a double-stranded intermediate composed of long strands of positive genomic RNA annealed to a complementary negative sense strand. The slight excess in positive-sense siRNAs in Aag2 cells and *A. aegypti* is likely to arise from Dcr2 recognition and cleavage of intrastrand secondary structures in the DENV2 genome. The distribution of viRNAs along the DENV2 genome in Aag2 cells at 5 days post DENV2 infection is relatively uniform, also implicating a long dsRNA replicative intermediate as the main source of DENV2-specific small RNAs in Aag2 mosquito cells. In DENV2-infected mosquitoes, the positive strand: negative strand ratio was even closer to 1∶1, with 55% of the DENV2-specific small RNAs being derived from the positive sense strand.

Previous studies of another flavivirus, WNV, in *Culex quinquefasciatus* mosquitoes showed that approximately 74% of the virus-specific small RNAs were from the positive sense RNA strand [43]. These differences in strand polarity ratios may be due to replication strategies of the viruses themselves or to a different RNAi response in *Culex* mosquitoes when compared to *A. aegypti*. *Culex* mosquitoes have a duplication of the *ago2* gene, which could result in differences in antiviral RNAi responses [37]. Small RNA deep sequencing of *A. aegypti* mosquitoes infected by the positive sense RNA alphavirus SINV showed that 54% of the virus-specific small RNAs were from the positive sense strand [45], a very similar proportion to our findings in DENV2-infected *A. aegypti*. When the alphavirus ONNV was studied in *A. gambiae* mosquitoes, the proportion of positive sense virus-specific small RNAs was slightly higher at 64% [45]. The differences seen between these alphavirus-infected mosquitoes also may be due to differences in SINV and ONNV replication mechanisms or due to different responses in the two mosquito genera.

The number of DENV2-specific small RNAs in our total RNA samples was very low. Next generation SOLiD sequencing revealed that that less than 0.02% of the small RNAs in the DENV2-infected Aag2 cell (5 dpi) library and less than 0.05% of the small RNAs in DENV2-infected *A. aegypti* mosquitoes (9 dpi) were DENV2-specific. These results appear to be typical of flavivirus-infected mosquitoes, as *Culex* mosquitoes infected with WNV had less than 0.05% WNV-specific small RNAs in the total small RNA population at 7 days post-infection and 0.12% WNV-specific small RNAs at 14 days post-infection [43]. This may be due to sequestration of flavivirus replication complexes in membrane-enclosed vesicles in mosquito as well as mammalian cells, preventing Dcr2 access to dsRNA replicative intermediates [40], [41], [50]. Alphavirus replication in mosquitoes appears to generate more virus-specific small RNAs. Approximately 10% of the 18–24 nt RNAs sequenced from SINV-infected *A. aegypti* mosquitoes were matches to the SINV genome [45]. Although in ONNV-infected *A. gambiae* mosquitoes the proportion of virus-specific small RNAs was lower, with 1.2% of the total small RNA reads matching the ONNV genome, still it was at least 10-fold higher than for any flavivirus reported to date [45]. These higher proportions of alphavirus small RNAs as compared to flavivirus small RNAs may be due to differences in accessibility of the replicative intermediate dsRNA to RNAi machinery during viral replication, or possibly because of more rapid viral replication to higher titers in alphavirus infected mosquitoes. Another reason for the increased numbers of alphaviral small RNAs in these studies may be that the mosquitoes were injected with SINV and ONNV, whereas infection of mosquitoes used in the DENV and WNV studies was established orally. Although we have presented clear evidence that RNAi plays an antiviral role against DENV2 [10], the low levels of DENV2 viRNAs in infected cells and mosquitoes raise the question whether the viRNAs themselves have an important role in the RNAi response. Possibly Dcr2 cleavage of replicating viral RNA alone helps to keep the DENV2 infection from overwhelming mosquito cells and causing excessive pathology and overt mortality in the insect.

The DENV2-specific viRNAs in both Aag2 cells (5 dpi) and *A. aegypti* mosquitoes were predominantly 21 nt long with similar proportions of sense and antisense polarities, suggesting that the underlying mechanistic aspects of their RNAi responses are similar. During our small RNA analysis we also discovered many CFAV-specific small RNAs in the Aag2 cell culture samples, but only a few CFAV-matching reads in the *A. aegypti* mosquitoes. The Aag2 cell line is persistently infected with this insect-only flavivirus, and it appears to activate the antiviral RNAi pathway. Although the CFAV RNA-specific proportion of small RNAs was higher (0.2–0.7%), the size distribution and polarity of the CFAV-specific small RNAs in Aag2 cells were similar to the DENV2-specific small RNAs found after DENV2-infection, and these characteristics suggest that they are products of the exogenous siRNA pathway. Possible effects of CFAV persistent infection on DENV2 replication in the Aag2 cells are unknown and need further study. The sequence identity between CFAV and DENV2 (Jamaica 1409 strain) RNAs is only 47%, so a sequence-specific response to DENV2 infection in CFAV-persistently infected Aag2 cells seems unlikely, although a change in level of RNAi activity due to persistent CFAV may have a non-specific effect on DENV2 replication in these cells.

In contrast to our findings for Aag2 cells, deep sequencing and analysis of small RNA in DENV2-infected C6/36 cells revealed abundant DENV2-specific small RNA that were longer than 21 nt and almost exclusively sense polarity, characteristics not expected of Dcr2-generated siRNAs. In addition, the C6/36 cell DENV2-specific small RNAs seemed to be generated only from specific regions of the genome. Further investigation is needed to determine if these correspond to secondary structures within the genome. Despite the greater numbers of virus-specific small RNAs in C6/36 cells, the overwhelming predominance of genome-sense small RNAs, even if they are loaded into a RISC, would result in inefficient cleavage of newly-synthesized viral genomes and a comparatively weak innate immune response. The lack of functional Dcr2 activity in C6/36 cells and production of predominantly positive-sense small RNAs may play a role in their increased ability to support the replication of arboviruses such as DENV and chikungunya virus, and may account for Igarashi's speculation that “the virus-sensitive C6/36 clone may lack efficient regulatory mechanism for virus RNA synthesis and virus production” [3].

The predominant length of DENV2-derived small RNAs in C6/36 cells was 27 nt, a size characteristic of piRNAs [28]. Production of piRNAs is Dcr1/Dcr2-independent and can be mediated by Ago3 [53]. Virus-specific piRNAs were recently described in *Drosophila* [36] and Zambon, et al. [55] showed that *piwi*-family mutants of *Drosophila* were more susceptible to Drosophila virus X infection. Logo analysis of DENV2- and CFAV-specific small RNAs from C6/36 cells showed a bias for adenine at the 10^th^ position from the 5′ end, which is also characteristic of piRNAs bound by Ago3 [35], [53].

We inadvertently co-infected the C6/36 cells with CFAV contained in the DENV2 stock, and the CFAV-specific small RNAs produced had similar properties to the DENV2-specific small RNAs, but were uncharacteristic of an exogenous siRNA pathway. The C6/36 cells also did not produce typical 21 nt viRNAs in response to infection by WNV, SINV or LACV (Brackney, et al., 2010 submitted). Earlier studies in C6/36 cells engineered to express dsRNA hairpin structures derived from DENV2 RNA showed small RNAs generated from these hairpins that migrated between 20 nt and 30 nt size markers, with a size appearing to be larger than 21 nt [56]. The cells expressing these inverted repeat transcripts were resistant to DENV2 infection, and in light of our current discovery of impaired Dcr2-like activity in C6/36 cells, we speculate that the increased resistance to DENV2 infection in this engineered cell line was probably due to a Dcr2-independent RNA silencing mechanism, such as the piRNA pathway.

In the study by Chotkowski et al. [8], northern blot hybridization using a sense-strand probe failed to detect WNV-specific siRNAs in C6/36 cells. If WNV-specific small RNAs were predominantly genome-sense, as in our study, they would be poorly detected by a positive-sense hybridization probe. Our *in vitro* assay indicated that C6/36 cells lack the ability to cleave long dsRNA into characteristic siRNAs. Only transfected siRNAs could be used to knock-down GFP expression from a plasmid in the cells, and long dsRNA did not. Although C6/36 cells appeared to lack efficient Dcr2 activity, addition of recombinant Dicer to the lysate resulted in production of siRNAs; it thus appeared that the lack of Dcr2 activity was not due to its inhibition in C6/36 cells. Northern blot analysis showed that *dcr2* was expressed at a somewhat reduced level in C6/36 cells compared to Aag2 cells; however, the magnitude of reduction does not appear to be sufficient to account for the lack of dicing activity. A recent study by Lim et al. [57] showed that missense mutations in the *Drosophila dcr2* DExH helicase domain or RNase III domain caused a loss of dsRNA processing activity. We have cloned and sequenced full-length *dcr2* cDNA from Aag2 cells and a 3920 nt fragment of C6/36 cell *dcr2* (equivalent of nt ∼1200-5120 on *A. aegypti dcr2*) (data not shown). The Aag2 *dcr2* nucleotide sequence was >99% identical to *A. aegypti dcr2*; however, the C6/36 *dcr2* fragment showed only 79% identity with the Aag2 full-length sequence. Translation of the nucleotide sequences revealed an apparent single nt deletion in C6/36 *dcr2* at nt 1508 that resulted in a termination codon, and thus a nonsense mutation. Our detection of full-length *dcr2* mRNA in C6/36 cells suggests that it does not undergo nonsense-mediated decay, as would be expected for early translation termination [58], so it is possible that a ribosomal frame-shift allows complete translation. However, because of the lack of availability of the authentic *A. albopictus* sequence and the high degree of divergence of C6/36 *dcr2* sequence from *A. aegypti dcr2*, we are unable to pinpoint particular mutations in C6/36 *dcr2* that could result in a change in phenotype.

The presence of unusual DENV-specific small RNAs in C6/36 cells coupled with ineffective Dcr2 activity suggested that a compensating mechanism is used by these cells for generation of viral-specific small RNAs. The piRNA pathway may serve as a backup mechanism when the exogenous siRNA pathway is not functioning correctly. Evidence for this hypothesis was seen when the endo-siRNA pathway was disrupted by mutation of *ago2* in *Drosophila*, resulting in the appearance of somatic cell piRNAs that possibly served as a backup in transposon surveillance [32], [59]. In our examination of RNAi in *A. gambiae* mosquitoes we found that co-injection into mosquitoes of dsRNA derived from the *ago3* sequence with ONNV resulted in increased ONNV titers, hinting at a possible redundant role for Ago3 in antiviral immunity in these mosquitoes [46]. Recently, viral small RNAs of various sizes other than 21 nt were found in a variety of animal cells infected with RNA viruses, suggesting roles for alternative RNA silencing pathways in antiviral defense [60].

In summary, we determined that DENV2-specific small RNAs produced during infection of *A. aegypti* mosquitoes and *A. aegypti* Aag2 mosquito cell cultures appear to be made via the exogenous siRNA pathway, but they are made in very low numbers, indicating that DENV2 may have a strategy to evade the antiviral RNAi response. *In vitro* studies demonstrated production of characteristic siRNA in Aag2 cells but indicated that C6/36 cells exhibit inefficient Dcr2 cleavage of long dsRNA. The C6/36 *A. albopictus* cell line produced more abundant DENV2-specific small RNAs, although they appeared to be generated by a different small RNA pathway, possibly through a piRNA-like mechanism, and this aberrant pattern of viral small RNA production extends to other flaviviruses, alphaviruses and bunyaviruses (Brackney, et al., 2010 submitted). The ability of C6/36 cells to support robust arbovirus replication may be due to lack of a complete, functional RNAi pathway. The evidence we have presented here indicates that C6/36 cells do not provide an accurate model for mosquito-arbovirus molecular interactions in the RNAi pathway.

## References

[1] Peleg J (1968). Growth of arboviruses in monolayers from subcultured mosquito embryo cells.. Virology.

[2] Singh KRP (1967). Cell Cultures Derived From Larvae Of *Aedes albopictus* (Skuse) and *Aedes aegypti* (L.).. Curr Sci.

[3] Igarashi A (1978). Isolation of a Singh's *Aedes albopictus* Cell Clone Sensitive to Dengue and Chikungunya Viruses.. J Gen Virol.

[4] Reigel F (1980). Studies on Igarashi's *Aedes albopictus* cell clone C6/36.. Experientia.

[5] Sasao F, Igarashi A, Fukai K (1980). Amino acid requirements for the growth of *Aedes albopictus* Clone C6/36 cells and for the production of dengue and chikungunya viruses in the infected cells.. Microbiology and Immunology.

[6] White LA (1987). Susceptibility of *Aedes albopictus* C6/36 cells to viral infection.. J Clin Microbiol.

[7] Vasilakis N, Deardorff ER, Kenney JL, Rossi SL, Hanley KA (2009). Mosquitoes Put the Brake on Arbovirus Evolution: Experimental Evolution Reveals Slower Mutation Accumulation in Mosquito Than Vertebrate Cells.. PLoS Pathog.

[8] Chotkowski HL, Ciota AT, Jia Y, Puig-Basagoiti F, Kramer LD (2008). West Nile virus infection of *Drosophila melanogaster* induces a protective RNAi response.. Virology.

[9] Cirimotich CM, Scott JC, Phillips AT, Geiss BJ, Olson KE (2009). Suppression of RNA Interference Increases Alphavirus Replication and Virus-Associated Mortality in *Aedes aegypti* Mosquitoes.. BMC Microbiol.

[10] Sánchez-Vargas I, Scott JC, Poole-Smith BK, Franz AW, Barbosa-Solomieu V (2009). Dengue virus type 2 infections of *Aedes aegypti* are modulated by the mosquito's RNA interference pathway.. PLoS Pathog.

[11] Lan Q, Fallon AM (1990). Small Heat Shock Proteins Distinguish between two Mosquito Species and Confirm Identity of Their Cell Lines.. Am J Trop Med Hyg.

[12] Galiana-Arnoux D, Dostert C, Schneemann A, Hoffmann JA, Imler J-L (2006). Essential function in vivo for Dicer-2 in host defense against RNA viruses in drosophila.. Nat Immunol.

[13] van Rij RP, Saleh M-C, Berry B, Foo C, Houk A (2006). The RNA silencing endonuclease Argonaute 2 mediates specific antiviral immunity in *Drosophila melanogaster*.. Genes & Development.

[14] Wang X-H, Aliyari R, Li W-X, Li H-W, Kim K (2006). RNA Interference Directs Innate Immunity Against Viruses in Adult Drosophila.. Science.

[15] Zamore PD, Haley B (2005). Ribo-gnome: The Big World of Small RNAs.. Science.

[16] Nykänen A, Haley B, Zamore PD (2001). ATP Requirements and Small Interfering RNA Structure in the RNA Interference Pathway.. Cell.

[17] Bernstein E, Caudy AA, Hammond SM, Hannon GJ (2001). Role for a bidentate ribonuclease in the initiation step of RNA interference.. Nature.

[18] Elbashir SM (2001). Duplexes of 21-nucleotide RNAs mediate RNA interference in cultured mammalian cells.. Nature.

[19] Elbashir SM, Martinez J, Patkaniowska A, Lendeckel W, Tuschl T (2001). Functional anatomy of siRNAs for mediating efficient RNAi in *Drosophila melanogaster* embryo lysate.. EMBO J.

[20] Liu Q (2003). R2D2, a bridge between the initiation and effector steps of the Drosophila RNAi pathway.. Science.

[21] Okamura K, Ishizuka A, Siomi H, Siomi MC (2004). Distinct roles for Argonaute proteins in small RNA-directed RNA cleavage pathways.. Genes Dev.

[22] Rand TA, Ginalski K, Grishin NV, Wang X (2004). Biochemical identification of Argonaute 2 as the sole protein required for RNA-induced silencing complex activity.. Proc Natl Acad Sci USA.

[23] Miyoshi K, Tsukumo H, Nagami T, Siomi H, Siomi MC (2005). Slicer function of Drosophila Argonautes and its involvement in RISC formation.. Genes & Development.

[24] Schwarz DS, Tomari Y, Zamore PD (2004). The RNA-Induced Silencing Complex Is a Mg2+-Dependent Endonuclease.. Current Biology.

[25] Schwarz DS, Hutvágner G, Haley B, Zamore PD (2002). Evidence that siRNAs Function as Guides, Not Primers, in the Drosophila and Human RNAi Pathways.. Molecular Cell.

[26] Aravin AA, Hannon GJ, Brennecke J (2007). The Piwi-piRNA pathway provides an adaptive defense in the transposon arms race.. Science.

[27] Vagin VV (2006). A distinct small RNA pathway silences selfish genetic elements in the germline.. Science.

[28] Saito K (2006). Specific association of Piwi with rasiRNAs derived from retrotransposon and heterochromatic regions in the Drosophila genome.. Genes Dev.

[29] Shpiz S, Kwon D, Rozovsky Y, Kalmykova A (2009). rasiRNA pathway controls antisense expression of Drosophila telomeric retrotransposons in the nucleus.. Nucl Acids Res.

[30] Okamura K, Balla S, Martin R, Liu N, Lai EC (2008). Two distinct mechanisms generate endogenous siRNAs from bidirectional transcription in *Drosophila melanogaster*.. Nat Struct Mol Biol.

[31] Chung W-J, Okamura K, Martin R, Lai EC (2008). Endogenous RNA Interference Provides a Somatic Defense against Drosophila Transposons.. Current Biology.

[32] Ghildiyal M, Seitz H, Horwich MD, Li C, Du T (2008). Endogenous siRNAs Derived from Transposons and mRNAs in Drosophila Somatic Cells.. Science.

[33] Horwich MD, Li C, Matranga C, Vagin V, Farley G (2007). The Drosophila RNA Methyltransferase, DmHen1, Modifies Germline piRNAs and Single-Stranded siRNAs in RISC.. Current Biology.

[34] Saito K (2007). Pimet, the Drosophila homolog of HEN1, mediates 2[prime]-O-methylation of Piwi- interacting RNAs at their 3[prime] ends.. Genes Dev.

[35] Gunawardane LS (2007). A slicer-mediated mechanism for repeat-associated siRNA 5′ end formation in Drosophila.. Science.

[36] Wu Q, Luo Y, Lu R, Lau N, Lai EC (2010). Virus discovery by deep sequencing and assembly of virus-derived small silencing RNAs.. Proceedings of the National Academy of Sciences.

[37] Campbell C, Black W, Hess A, Foy B (2008). Comparative genomics of small RNA regulatory pathway components in vector mosquitoes.. BMC Genomics.

[38] Li S, Mead E, Liang S, Tu Z (2009). Direct sequencing and expression analysis of a large number of miRNAs in *Aedes aegypti* and a multi-species survey of novel mosquito miRNAs.. BMC Genomics.

[39] Skalsky R, Vanlandingham D, Scholle F, Higgs S, Cullen B (2010). Identification of microRNAs expressed in two mosquito vectors, *Aedes albopictus* and *Culex quinquefasciatus*.. BMC Genomics.

[40] Uchil PD, Satchidanandam V (2003). Architecture of the Flaviviral Replication Complex: Protease, Nuclease, and Detergents Reveal Encasement Within Double-Layered Membrane Compartments.. J Biol Chem.

[41] Welsch S, Miller S, Romero-Brey I, Merz A, Bleck CKE (2009). Composition and Three-Dimensional Architecture of the Dengue Virus Replication and Assembly Sites.. Cell Host Microbe.

[42] Stollar V, Stollar BD (1970). Immunochemical Measurement of Double-stranded RNA of Uninfected and Arbovirus-Infected Mammalian Cells.. Proceedings of the National Academy of Sciences of the United States of America.

[43] Brackney DE, Beane JE, Ebel GD (2009). RNAi Targeting of West Nile Virus in Mosquito Midguts Promotes Virus Diversification.. PLoS Pathog.

[44] Campbell CL, Keene KM, Brackney DE, Olson KE, Blair CD (2008). *Aedes aegypti* uses RNA interference in defense against Sindbis virus infection.. BMC Microbiol.

[45] Myles KM, Wiley MR, Morazzani EM, Adelman ZN (2008). Alphavirus-derived small RNAs modulate pathogenesis in disease vector mosquitoes.. Proceedings of the National Academy of Sciences.

[46] Keene KM, Foy BD, Sanchez-Vargas I, Beaty BJ, Blair CD (2004). RNA interference acts as a natural antiviral response to O'nyong-nyong virus (*Alphavirus*; *Togaviridae*) infection of Anopheles gambiae.. Proceedings of the National Academy of Sciences of the United States of America.

[47] Haley B, Tang G, Zamore P (2003). In vitro analysis of RNA interference in *Drosophila melanogaster*.. Methods.

[48] Schneider TD, Stephens RM (1990). Sequence logos: a new way to display consensus sequences.. Nucl Acids Res.

[49] Crooks GE, Hon G, Chandonia J-M, Brenner SE (2004). WebLogo: A Sequence Logo Generator.. Genome Research.

[50] Miller S, Kastner S, Krijnse-Locker J, Bühler S, Bartenschlager R (2007). The Non-structural Protein 4A of Dengue Virus Is an Integral Membrane Protein Inducing Membrane Alterations in a 2K-regulated Manner.. J Biol Chem.

[51] Cammisa-Parks H, Cisar LA, Kane A, Stollar V (1992). The complete nucleotide sequence of cell fusing agent (CFA): Homology between the nonstructural proteins encoded by CFA and the nonstructural proteins encoded by arthropod-borne flaviviruses.. Virology.

[52] Stollar V, Thomas VL (1975). An agent in the *Aedes aegypti* cell line (Peleg) which causes fusion of *Aedes albopictus* cells.. Virology.

[53] Brennecke J, Aravin AA, Stark A, Dus M, Kellis M (2007). Discrete Small RNA-Generating Loci as Master Regulators of Transposon Activity in Drosophila.. Cell.

[54] Stollar V, Schlesinger RW, Stevens TM (1967). Studies on the nature of dengue viruses: III. RNA synthesis in cells infected with type 2 dengue virus.. Virology.

[55] Zambon RA, Vakharia VN, Wu LP (2006). RNAi is an antiviral immune response against a dsRNA virus in *Drosophila melanogaster*.. Cellular Microbiology.

[56] Adelman ZN, Sanchez-Vargas I, Travanty EA, Carlson JO, Beaty BJ (2002). RNA silencing of dengue virus type 2 replication in transformed C6/36 mosquito cells transcribing an inverted-repeat RNA derived from the virus genome.. J Virol.

[57] Lim DH, Kim J, Kim S, Carthew RW, Lee YS (2008). Functional analysis of dicer-2 missense mutations in the siRNA pathway of Drosophila.. Biochemical and Biophysical Research Communications.

[58] Harigaya Y, Parker R (2010). No-go decay: a quality control mechanism for RNA in translation.. Wiley Interdisciplinary Reviews: RNA.

[59] Ghildiyal M, Zamore PD (2009). Small silencing RNAs: an expanding universe.. Nat Rev Genet.

[60] Parameswaran P, Sklan E, Wilkins C, Burgon T, Samuel MA (2010). Six RNA Viruses and Forty-One Hosts: Viral Small RNAs and Modulation of Small RNA Repertoires in Vertebrate and Invertebrate Systems.. PLoS Pathog.

